# A Case of Well-Differentiated Endometrial Adenocarcinoma in a Postmenopausal Woman

**DOI:** 10.7759/cureus.61070

**Published:** 2024-05-25

**Authors:** Siddhi Shringi, Anil K Agrawal, K.M. Hiwale, Pravin Gadkari

**Affiliations:** 1 Pathology, Jawaharlal Nehru Medical College, Datta Meghe Institute of Higher Education and Research, Wardha, IND

**Keywords:** histopathological examination, hysterectomy, abnormal vaginal bleeding, pelvic pain, postmenopausal bleeding, endometrial adenocarcinoma

## Abstract

Endometrial adenocarcinoma is a prevalent malignancy among postmenopausal women, often presenting with symptoms such as abnormal vaginal bleeding and pelvic pain. We present a case of a 60-year-old postmenopausal female who exhibited abnormal vaginal bleeding for three months, accompanied by pelvic pain and unintentional weight loss. Clinical evaluation, including physical examination, imaging studies, and histopathological examination, led to the diagnosis of well-differentiated endometrial adenocarcinoma. The patient underwent an abdominal hysterectomy with bilateral salpingo-oophorectomy, and histopathological analysis confirmed invasive tumor involvement in the lower uterine segment and cervix. The final pathological tumor, node, and metastasis (TNM) staging was reported as pT1b No Mx, FIGO (International Federation of Gynecology and Obstetrics) stage II. This case underscores the importance of considering endometrial adenocarcinoma in the differential diagnosis of postmenopausal bleeding and highlights the significance of timely diagnosis and multidisciplinary management for optimizing patient outcomes.

## Introduction

Endometrial cancer is the most common gynecologic malignancy in developed countries, with its incidence steadily rising worldwide [1]. Postmenopausal bleeding is a hallmark symptom, often prompting clinical evaluation for endometrial pathology [2]. While most cases of postmenopausal bleeding are benign, up to 10% can be attributed to endometrial malignancies, necessitating thorough investigation [3]. Risk factors for endometrial cancer include obesity, nulliparity, late menopause, unopposed estrogen therapy, and diabetes mellitus [4]. Endometrial cancer can be classified into two major histologic types: type I, which includes endometrioid adenocarcinoma, and type II, which encompasses serous and clear cell carcinomas [5]. Type I tumors are typically estrogen-dependent, associated with endometrial hyperplasia, and present at an early stage with favorable outcomes [6]. On the other hand, type II tumors are estrogen-independent, high-grade lesions, often diagnosed at an advanced stage, with a poorer prognosis [7].

Diagnosis of endometrial cancer relies on a combination of clinical evaluation, imaging studies, and histopathological examination. Transvaginal ultrasound is often used as the initial imaging modality, demonstrating endometrial thickening as a common finding [8]. Histopathological examination of endometrial biopsy specimens remains the gold standard for definitive diagnosis, with endometrial sampling demonstrating high sensitivity and specificity [9]. Once diagnosed, the management of endometrial cancer depends on several factors, including tumor stage, grade, histology, and patient comorbidities. Early-stage disease is often treated with surgery, typically hysterectomy with bilateral salpingo-oophorectomy, offering a potential cure [10]. Adjuvant therapy, including radiotherapy and chemotherapy, may be considered in cases of advanced disease or high-risk features [11]. The prognosis for endometrial cancer is generally favorable, especially for early-stage disease. However, recurrence rates vary depending on tumor characteristics and treatment modalities [12]. Close surveillance following treatment is essential to detect recurrent disease early and optimize patient outcomes.

## Case presentation

A 60-year-old postmenopausal female presented to the gynecology clinic with a chief complaint of abnormal vaginal bleeding persisting for the past three months. Initially mild, the bleeding had progressively worsened over the recent weeks, prompting her visit. She described experiencing postmenopausal bleeding for the first time, accompanied by intermittent pelvic pain localized primarily to the lower abdomen. The pain was moderate in intensity and occurred intermittently. Additionally, she reported an unintentional weight loss of approximately 10 kg over the past two months despite no significant changes in her diet or physical activity levels. Her medical history was notable for controlled hypertension, managed with antihypertensive medications. She had undergone menopause at the age of 50 years and had no prior history of abnormal uterine bleeding or gynecological malignancies.

Upon physical examination, the patient appeared pale and reported easy fatigability. Vital signs were within normal limits. Abdominal examination revealed mild tenderness upon palpation of the lower abdomen, with no palpable masses or organomegaly appreciated. Speculum examination revealed scant vaginal bleeding, and no obvious cervical lesions were visualized. Bimanual pelvic examination elicited bilaterally tenderness in the uterine adnexal regions, with no palpable masses appreciated. Further evaluation included transvaginal ultrasound, which revealed a thickened endometrial stripe measuring 15 millimeters with no obvious focal lesions or uterine abnormalities noted. Considering the clinical presentation and imaging findings, an endometrial biopsy was performed. Histopathological examination revealed well-differentiated adenocarcinoma. Additional laboratory investigations were within normal limits, including a complete blood count, liver function, and renal function tests. However, cancer antigen 125 (CA-125) tumor marker levels were mildly elevated at 42 U/ml (normal range: 0-35 U/ml).

**Figure 1:**
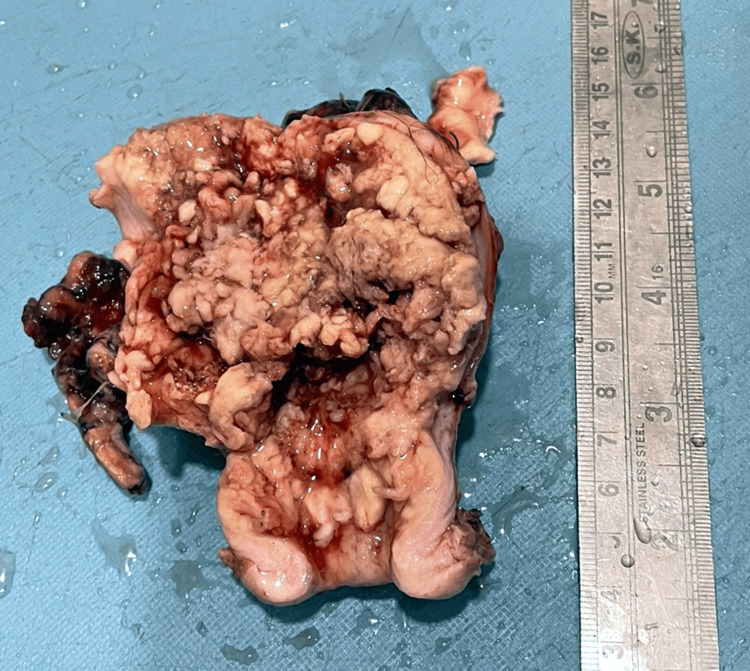
A single, well-circumscribed, friable, exophytic growth obliterating the endometrial cavity, measuring 8.5 x 7 x 1.5 cm.

The clinical presentation, imaging findings, and histopathological examination results established a diagnosis of well-differentiated adenocarcinoma. Consequently, the patient underwent abdominal hysterectomy with bilateral salpingo-oophorectomy. Upon gross examination, the excised specimen revealed a single, well-circumscribed, friable, exophytic growth, obliterating the endometrial cavity, measuring 8.5 x 7 x 1.5 cm (Figure 1). The growth appeared to infiltrate the lower uterine segment and the endocervical stroma. However, bilateral fallopian tubes and ovaries were grossly not involved by the tumor.

**Figure 2:**
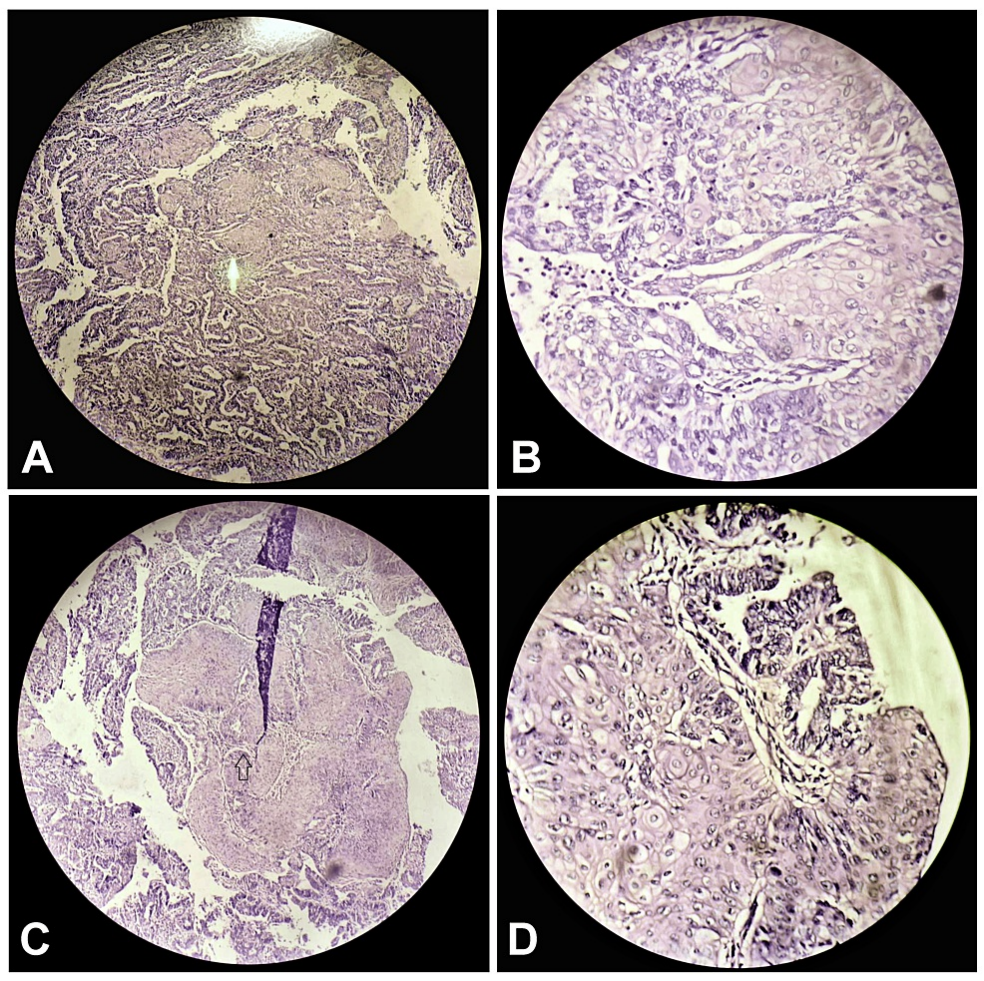
Histological examination shows well-differentiated adenocarcinoma of endometrioid type I at (A) 10x magnification, (B) 40x magnification, (C) 10x magnification, and (D) 400x magnification.

Histological examination of the gross specimen confirmed the presence of well-differentiated endometrioid adenocarcinoma (type I) with more than 50% invasion into the myometrium (Figure 2). The tumor mass infiltrated into the lower uterine segment and cervix. The final pathological tumor, node, and metastasis (TNM) staging was reported as pT1b No Mx, FIGO (International Federation of Gynecology and Obstetrics) stage II, indicating localized disease with invasion into the myometrium but no evidence of distant metastasis.

## Discussion

Endometrial adenocarcinoma is the most common gynecologic malignancy in developed countries, with an estimated annual incidence of 65,620 new cases and 12,590 deaths in the United States alone [13]. It typically affects postmenopausal women, as seen in the presented case. The etiology of endometrial adenocarcinoma is multifactorial, with obesity, unopposed estrogen therapy, nulliparity, late menopause, and diabetes mellitus identified as significant risk factors [14]. The clinical presentation of endometrial adenocarcinoma often includes abnormal vaginal bleeding, pelvic pain, and weight loss, consistent with the symptoms observed in the patient. Abnormal vaginal bleeding, particularly in postmenopausal women, warrants thorough investigation to rule out endometrial pathology, as it may indicate malignancy. The presence of pelvic pain and unintentional weight loss further raises suspicion of an underlying malignancy, prompting a comprehensive diagnostic workup [15].

Diagnostic evaluation typically involves a combination of imaging studies, such as transvaginal ultrasound and magnetic resonance imaging (MRI), and histopathological examination of endometrial biopsy specimens. Transvaginal ultrasound is a valuable tool for assessing endometrial thickness and detecting focal lesions, aiding in the diagnosis of endometrial adenocarcinoma [16]. In the presented case, transvaginal ultrasound revealed a thickened endometrial stripe, prompting further investigation. Histopathological examination remains the gold standard for diagnosing endometrial adenocarcinoma. Endometrial biopsy allows for the assessment of tissue architecture and the identification of malignant cells. Additionally, tumor marker assessment, including CA-125, may aid in diagnosing and monitoring disease progression. In the presented case, histopathological examination of the endometrial biopsy specimen confirmed the presence of well-differentiated adenocarcinoma, with mildly elevated CA-125 levels further supporting the diagnosis [17].

Treatment options for endometrial adenocarcinoma depend on the stage of the disease and may include surgery, chemotherapy, and radiotherapy. Surgical management, such as hysterectomy with bilateral salpingo-oophorectomy, is often recommended for early-stage disease. In the presented case, the patient underwent abdominal hysterectomy with bilateral salpingo-oophorectomy, aiming to achieve complete resection of the tumor and prevent disease progression [18]. The prognosis for endometrial adenocarcinoma is generally favorable, particularly for early-stage disease. However, advanced-stage disease and specific histological subtypes, such as serous and clear cell carcinoma, may carry a poorer prognosis [19]. Therefore, early diagnosis and appropriate management are crucial for optimizing outcomes and improving survival rates in patients with endometrial adenocarcinoma.

## Conclusions

In conclusion, the presented case underscores the importance of maintaining a high index of suspicion for endometrial adenocarcinoma in postmenopausal women presenting with abnormal vaginal bleeding, pelvic pain, and unintentional weight loss. Early diagnosis through a comprehensive diagnostic workup, including imaging studies and histopathological examination, is paramount for the timely initiation of appropriate management. Surgical intervention, such as hysterectomy with bilateral salpingo-oophorectomy, remains a cornerstone of treatment for early-stage disease, with favorable prognostic implications. Multidisciplinary collaboration involving gynecologists, oncologists, and pathologists is essential for optimizing outcomes and improving survival rates in patients with endometrial adenocarcinoma. Continued research efforts to elucidate the molecular mechanisms underlying endometrial carcinogenesis and identify novel therapeutic targets are warranted to advance the management of this prevalent gynecologic malignancy.
